# Exploring the Effects of the COVID-19 Pandemic on Mental Health and Well-Being of Migrant Populations in Europe: An Equity-Focused Scoping Review

**DOI:** 10.3390/bs12100393

**Published:** 2022-10-14

**Authors:** Violeta Alarcão, Ana Virgolino, Miodraga Stefanovska-Petkovska, Júlia Neves

**Affiliations:** 1 Centro de Investigação e Estudos de Sociologia, Iscte—Instituto Universitário de Lisboa, Avenida das Forças Armadas, 1649-026 Lisboa, Portugal; 2 Instituto de Saúde Ambiental, Faculdade de Medicina, Universidade de Lisboa, Avenida Professor Egas Moniz, 1649-028 Lisboa, Portugal

**Keywords:** coronavirus disease 2019, mental health and well-being, social determinants of health, migration

## Abstract

The pandemic is aggravating health inequalities, particularly mental health inequalities, while revealing the social determinants of these inequalities, including migration as a social determinant that mediates the interaction of social, economic, cultural, institutional, and structural factors with health indicators. Therefore, it is of most relevance to identify the multiple interconnected factors that influence the mental health and well-being of migrant populations. A scoping review was developed to map the research performed in this area and to identify any gaps in knowledge, following the PRISMA extension for scoping reviews. MEDLINE, Scopus, and WHO Global Health research databases on COVID-19 were searched from January 2020 to October 2021. The review followed the inclusion criteria Population/Concept/Context (PCC): Population-Adult International migrants (including refugees, asylum seekers, and undocumented migrants); Concept-determinants of (and factors influencing) mental health and well-being; Context-COVID-19 anywhere in the world. Of the sixty-five selected studies, eleven were from European countries and were the focus of this review with special attention to health inequalities experienced by migrants in Europe. The results cover a diversity of themes related to the effects of COVID-19 on the mental health of migrants (country-level environmental factors, social determinants of mental health, mental health indicators and outcomes), responses (such as solidarity and resilience), populations, and study methods. The evidence found can inform recommendations and interventions focused on health promotion and mitigation of the inequalities accentuated by the pandemic.

## 1. Introduction

As rapid as the COVID-19 pandemic emerged, it gave rise to a series of disruptive consequences in the economic, health, and educational sectors that contributed to a scenario of disproportional economic and social vulnerability. These effects of the pandemic seem to be greater in socially vulnerable and marginalized groups, more specifically migrant populations. While these groups are overrepresented in COVID-19 cases in terms of laboratory diagnosis, hospital admissions, intensive care, and death statistics in all countries according to the available data [1,2,3,4], they continue to face structural barriers to COVID-19 control efforts, such as testing and vaccination [5,6]. As a result, in addition to the adverse effects on their physical health, they are prone to the development of mental health and well-being ill-effects, thus positioning them as one of the most vulnerable and neglected groups in the COVID-19 pandemic [7,8,9].

This research centralizes around the premise that the pandemic is aggravating health inequalities, particularly mental health inequalities, while revealing the social determinants of these inequalities, including migration as a determinant that mediates the interaction of social, economic, cultural, institutional, and structural factors with health indicators, affecting the different spheres of life [10,11,12,13]. Therefore, it is relevant and necessary to identify the multiple factors that interconnect and influence mental health and well-being to understand the interdependencies between mental health and global challenges, such as the current context and impacts at the individual and societal levels.

Intersectional approaches are needed to analyze the complexity of the pandemic situation and adapt the political responses to deal with this complexity. Intersectionality is a theoretical-methodological powerful tool to reveal processes of interaction between power relations and categories—such as gender, class, race, and ethnicity—in individual, collective, and cultural/institutional contexts, yet still with limited use in mental health. Using an intersectional lens of analysis can help to identify differentials in the mental health effects of the pandemic between individuals and social groups, facilitating the elaboration of political responses tailored to people at the most marginalized intersections (racial/ethnic minorities, women, and undocumented workers) with a focus on equity [14,15,16,17].

This scoping review aims to identify and summarize the existing research on the effects of the COVID-19 pandemic on the mental health of migrants with reflection on the European context (an under-researched region needing a focused analysis) to inform suited recommendations for migrant-sensitive and migrant-inclusive healthcare. The overarching research question was to pinpoint the multiple factors influencing the COVID-19 pandemic’s effects on the mental health of international adult migrants (including refugees, asylum seekers, and irregular migrants).

## 2. Methods

### 2.1. Study Design

In early October 2021, we conducted a preliminary search on the Joanna Briggs Institute (JBI) Database of Systematic Reviews and Implementation Reports and the Cochrane Database of Systematic Reviews for identifying existing scoping and systematic reviews on the topic. No studies on the intersections between COVID-19, mental health, and migrants were found. Therefore, a scoping review was developed to map the research carried out in this area and to identify existing knowledge gaps, since scoping reviews provide a comprehensive overview to address broader review questions than traditionally more specific systematic reviews of effectiveness or qualitative evidence [18].

The protocol was registered with the Open Science Framework on 5 November 2021. The review was undertaken following the Preferred Reporting Items for Systematic Reviews and Meta-Analyses extension for scoping reviews—PRISMA-ScR [19] and the recommendations made by the JBI, which is a global organization promoting and supporting evidence-based decisions that improve health and health service delivery [18].

### 2.2. Search Strategy

A systematic literature search was performed using the following electronic databases: MEDLINE (via PubMed), Scopus, and WHO Global Health research database on COVID-19, using combinations of the search terms, tailored to the syntax, and functionality of each database. All searches were conducted on 13 October 2021 and tailored to each electronic database (See the example search for MEDLINE in Appendix A). A date range limitation of ‘2020—current’ was defined, and only English-written documents were considered eligible for inclusion.

### 2.3. Inclusion and Exclusion Criteria

The International Organization for Migration (IOM) defines a migrant as “any person who is moving or has moved across an international border or within a State away from his/her habitual place of residence, regardless of (1) the person’s legal status; (2) whether the movement is voluntary or involuntary; (3) what the causes for the movement are; or (4) what the length of the stay is”) was considered [20]. So, articles in which the population of interest did not meet the criteria stated in this definition were excluded.

The PCC method (Population (or participants)/Concept/Context) recommended by the JBI to identify the main concepts in the primary review questions was used for the search strategy and the definition of inclusion criteria [18]: P (Population = International migrant, Refugee, Asylum seeker, Undocumented migrant, age 18 and above), C (Concept = empirical data on individual, social, cultural, institutional, and structural factors influencing mental health and well-being outcomes), C (Context = Studies on any country in the world reporting international migrants’ mental health and well-being during the COVID-19 pandemic). Only empirical studies were included, regardless of research design, sample size, and methods used. An exception was intervention studies, which were excluded, because of their distinguished features compared to observational studies. Book reviews, book chapters, conference proceedings, editorials, commentaries, guidelines, study protocols, and vignette studies were also excluded. Each relevant record was reviewed independently by two authors, which screened titles and abstracts, and when needed, full texts. A final decision was obtained for each record, with uncertainties and disagreements resolved in consultation with a third author.

### 2.4. Data Extraction and Analysis

For all the articles included in the final analysis, data were extracted by two of the authors and included the following variables: (1) Author(s) and year of publication, (2) country, (3) population (sample size, type of migrant, country of origin, age, gender), (4) aim, (5) design, (6) mental health outcome(s), (7) social determinants of health, (8) overall results, (9) overall limitations, (10) and overall recommendations. Bibliometric data regarding the journal’s title, publication quartile, and domain/area of work (i.e., the area with the highest quartile in the year of the study publication according to the Scimago Journal and Country Rank) were also collected. Methodological quality or risk of bias of the included articles was not appraised because it was not relevant to the scoping review objectives, which is consistent with guidance on scoping review conduct [18]. Results were synthesized using a thematic approach focusing on the identification of relevant themes related to individual, social, cultural, institutional, and structural factors influencing mental health outcomes.

## 3. Results

**Figure 1:**
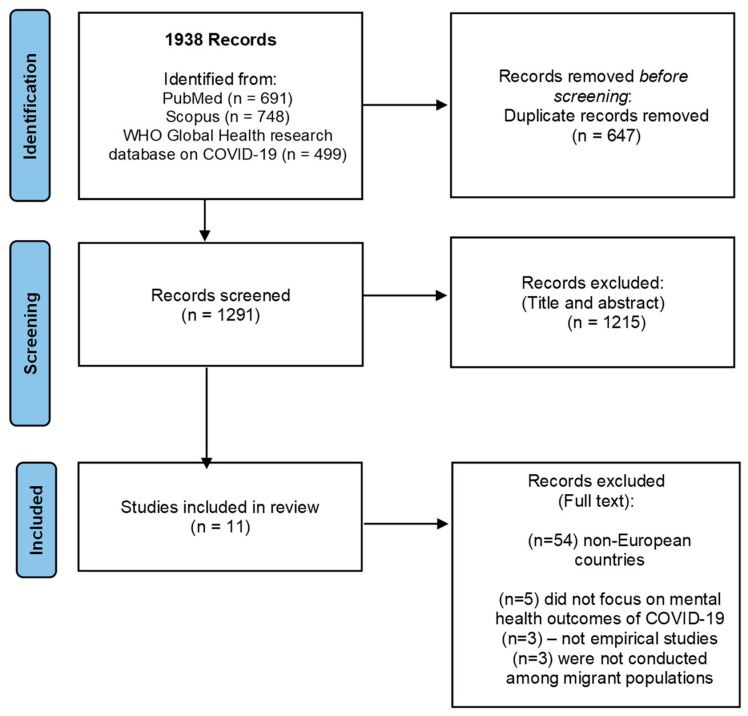
PRISMA 2020 flow diagram.

A total of 1938 publications were identified from the three searched databases. Once duplicates were removed, 1291 articles remained for screening. Of these, 1226 were excluded because they were not empirical studies, did not focus on mental health outcomes, or were not conducted among migrant populations. As a result, 65 studies could be included. Eleven articles (17%) were conducted in European countries and were specifically selected to be included in this review paper for mapping the research on the effects of the COVID-19 pandemic on the mental health of migrants as well as to identify knowledge gaps in Europe. A flow diagram detailing the number of studies included and excluded at each stage for this review is provided in Figure 1.

### 3.1. Characteristics of Selected Articles

All of the selected studies were published in journals with impact factor, mainly in journals ranked in the first quartile (7/11). Three studies were published in journals in the second quartile, and one study in the third quartile. In terms of the subject area, four articles were published in the Public health, Environmental and Occupational Health domain, two in Psychiatry and mental health, two in Geography, Planning and Development, and one in Clinical psychology, in Leadership and Management, and Health, Toxicology and Mutagenesis.

The studies were conducted in eight European countries and one publication was from a European Consortium, the ApartTogether Consortium, led by the Ghent University in Belgium and the University of Copenhagen in Denmark, and the Migration and Health program of the WHO Regional Office for Europe [21].

Two of the eleven studies were conducted in Italy with migrant patients in mental health outpatient departments, one published in 2020 aiming to evaluate the impact of the COVID-19–related lockdown on the difficulties in the utilization of mental health services and follow-up adherence [22], and the other published in 2022 to investigate the effects on the mental health outcomes [23].

Two other studies were conducted in the United Kingdom (UK), one with Black, Asian, and Minority Ethnicity group (BAME) healthcare workers [24], and another with data from the UK Household Longitudinal Survey and the first two waves (April and May 2020) of the COVID-19 survey via web-based questionnaires applied to UK-born and foreign-born men [25].

Additionally, two studies were related to the Netherlands—more specifically an online survey among Chinese immigrants living in the Netherlands [26] and a Polish study of three population groups: non-immigrants living in Poland, Dutch citizens, and Polish immigrants living in the Netherlands [27].

Finally, four countries (Spain, Switzerland, France, and Germany) had one publication each, with different populations and approaches. Research conducted in Spain was centered on the role of resilience for mental health in times of COVID-19 across groups of migration and non-migration individuals [28]. The study in Switzerland was conducted during the COVID-19 lockdown in April–May 2020 with undocumented and recently regularized migrants [29], while the research in France focused on the changes in mental health among disadvantaged immigrants from Sub-Saharan Africa in the greater Paris area with data collected before and during the first COVID-19-related lockdown [30]. The investigation yield in Germany relies on a cohort of outpatients at a psychiatric outpatient clinic with a high percentage of patients with a migration background [31].

The studies used a quantitative approach, with online and/or telephone surveys. There is a diversity in the mental health outcomes analyzed, from depression, anxiety, and stress, to sleeping problems and substance use, satisfaction with life, and responses (resilience, and solidarity, among other strategies).

Table 1 presents more details on the characteristics of the studies.

### 3.2. Country-Level Environment

The COVID-19 pandemic has posed new challenges to societies and their health systems. The included papers mention a series of unprecedented interventions implemented, from minor (mask wearing and hand hygiene) to major readjustments to everyday life, such as the “lockdown” periods that first took place in many countries between March and June 2020 to reduce the spread of SARS-CoV-2 infection [31], with Italy being the first European country to be brutally impacted [23].

Preventive actions involved a series of escalating restrictions on everyday life, including limitations on gatherings, travel restrictions, and minimization of movement outside of the house, with several services closed and social activities suspended. In addition to the tremendous consequences to the economy, socio-economic inequalities were exacerbated during the lockdown. The United Kingdom is among the countries where pre-existing socioeconomic inequalities have become worse than before the pandemic due to the adopted measures [25].

Switzerland was among the countries most rapidly and severely affected relative to its size, with a peak incidence of new positive tests on 30 March 2020, in Geneva, where undocumented migrants represent about 2.5% of the 500,000 residents. The duration of the public health lockdown measures put migrants, especially undocumented migrants, at increased risk of not being able to meet their basic needs [29]. While strongly exposed to the disease, some migrants did not have sufficient information about the pandemic and policy measures. This was the case in France, even with information campaigns launched to address the specific needs of this population [30]. Data from the French National Institute of Statistics revealed that the mortality rate was twice as high among immigrants as among natives during the first wave of the COVID-19 pandemic in April and March, and among immigrants from sub-Saharan Africa, excess mortality was estimated at +114% [30].

In addition to social isolation, COVID-19 has demanded people adapt to novel situations and future uncertainty, which represented a great psychological burden. A study conducted in Poland and the Netherlands to explore environmental stress and the quality of life connected with COVID-19 has shown cultural differences between the two countries, that can be linked to the government’s crisis management in these countries [27]. Anxiety decreased in the group of native Dutch citizens during the pandemic, possibly due to the Dutch government’s positive reinforcement (e.g., the police praised citizens for keeping a distance of 1.5 m, and fewer bans). Moreover, the sense of the quality of life increased during the pandemic among Polish immigrants, even though they had the highest intensity of fear and negative feelings. On the contrary, anxiety increased among Poles living in Poland, which may result from more rigid restrictions (e.g., wearing masks, introducing various bans, or high fines for non-compliance with the rules). Another explanation can be the efficiency of healthcare in the Netherlands, while planned visits to specialists were suspended in Poland [27].

### 3.3. Social Determinants of Mental Health

- Economic stability (employment, income, housing, food security)

Of the 11 articles selected, six presented findings on economic and labor instability in migrant populations during COVID-19 [21,23,24,25,29,30].

Socioeconomic issues linked to unemployment and immigration status uncertainty increased the risk of mental suffering due to the pandemic [23]. COVID-19 has been impacting disproportionally the most socially vulnerable groups, who are at higher risk of experiencing negative mental health outcomes. Migrants reporting most challenges in securing material and medical daily needs, such as housing, work, food, and clothes also reported more negative mental health outcomes, including more feelings of anxiety and depression [21,30].

Most migrants work in jobs (domestic or construction work) requiring their presence, which added to their dense housing conditions, puts them at higher risk of infection than the general population, across several settings [24,29,30]. Findings from the UK Understanding Society COVID-19 longitudinal survey showed that migrant working men were more economically affected than their native-born counterparts, namely in terms of losing work hours, and that this widening of the social and economic divisions was followed by an increasing gap in mental well-being [25].

- Socio-demographic characteristics of the migrant populations (gender, age, ethnicity, education)

Gender differences in health outcomes in the context of COVID-19 were observed in the selected studies. Moran and collaborators concluded that while men have a higher risk of COVID-19 mortality and severity, the indirect consequences of the virus tend to impact more women, due to their greater socioeconomic vulnerability. Impacts range from job and income losses to increasing gender-based violence and mental health. However, men who are marginalized or in disadvantaged positions are also particularly vulnerable [31].

Aragona et al. presented the negative impacts of the COVID-19 lockdown on access to mental health services and illustrated how the pre-pandemic situation of vulnerability put some subgroups of the population more at risk, including people with lower economic status, homelessness, migrants, and patients with mental health disorders. Additionally, some individuals fall into several of these subgroups, thus facing a significantly higher risk of negative mental health consequences. In Italy, there are increasing cases of asylum seekers whose claim has been rejected that are without documents, without jobs, homeless, and living in poverty. Those most vulnerable migrants also often have symptoms of post-traumatic stress disorder (PTSD) due to their migratory experience, as well as depressive and adjustment disorders [22].

In the ApartTogether online global survey of refugees and migrants, individuals with citizenship reported the least effect of COVID-19 on their mental health, compared to the most vulnerable groups of migrants (i.e., those living in asylum centers or on the street, those without documents or with temporary documents). Younger participants also reported a less negative effect of COVID-19 on their anxiety and depression, indicating an age effect [21].

- Healthcare access and treatment compliance

Migrants more frequently face limited access to medical care, diminishing the odds of seeking medical treatment early enough to reduce the number of undetected cases and self-isolate at home, which may accelerate the transmission of the disease. In addition, during the COVID-19 pandemic, access to public hospitals has been controlled by security staff, with the likely consequence to repel undocumented migrants. Data since the beginning of the COVID-19 crisis indicates that undocumented migrants have lower utilization of healthcare, compared to regularized migrants. Additionally, poor mental health was related to the avoidance of health care [22].

During the lockdown, patients reported barriers to mental health care access (although psychiatric care remained available online). This perceived unavailability of services due to the COVID-19 emergency is not only related to a reduction in the number of patients and follow-up visits but also influenced treatment discontinuation [22,23]. Aragona et al., in their study of migrants in treatment at an outpatient department, reported a 32% discontinuation rate in the psychopharmacological treatment and 52% in psychotherapy treatment [23].

Additionally, the study in France has shown that the specific policy measures implemented by the French government, such as the validity of all residence permits and the State Medical Aid (health insurance for undocumented persons), were only acknowledged by a few migrants. However, regarding strategies to preserve one’s health, a majority mentioned using barrier measures (masks, hand washing, physical distancing) [30].

### 3.4. Mental Health Indicators and Outcomes

- Mental and family-related distress

Among individuals already in treatment before the pandemic, there was an increase in the frequency of psychic anxiety, depressive symptoms, feelings of tension/nervousness, and sleep disorders, ranging between 74% and 91%, and including anxiety specifically related to the coronavirus (e.g., fear of contamination). In terms of the effects of the lockdown, mental symptoms were unchanged or improved in many patients. However, anxiety and nervousness were reported as the most frequently worsened symptoms, but never above 50% [23].

A study of minority ethnic healthcare workers has shown feelings of anxiety about the working condition and about family, particularly living in social isolation during COVID-19, with a significant impact on their mental health [24].

A study with female psychiatric patients also has found greater family-related distress, including more household tension, more housework, and more domestic violence, especially among those with a migration background [31].

- Stigmatization and discrimination of vulnerable groups

Since the earliest cases were reported in China, several studies have drawn attention to the rise of stigmatization and racism against Chinese and Asian immigrants. As the study conducted among Chinese immigrants in the Netherlands has shown, the pandemic and its societal dynamics harmed migrants’ views of their host country. Besides feelings of fear of COVID-19, financial consequences, social isolation, feelings of lost time, missing China, and perceived travel restrictions to China, more experiences of racism were frequently reported [26].

Other results have shown that refugees and migrants with higher perceived discrimination levels since the pandemic also reported worse mental health outcomes [21].

### 3.5. Coping Behaviors

The German study evaluating the experience of the pandemic among a cohort of outpatients at a large psychiatric outpatient institution measured the level of distress concerning COVID-19 lockdown and found increased concern about relatives, anxiety, worse sleep, and more physical symptoms, with a small group also reporting increased drug consumption. Women with a migrant background showed greater lockdown-related stress [31].

Meanwhile, the Spanish study examining the relationship between mental health and resilience across groups of migration and non-migration found a moderating group effect with higher resilience among individual migrants. This may be because migrants have a larger repertoire of coping strategies to deal with stressful situations such as the COVID-19 restrictions [28].

Due to the significant difficulties to fulfill their financial, material, and food needs during the pandemic, migrants engaged in seeking external support, such as using the strategy of requesting a financial loan from the immediate social circle of friends or relatives [29].

## 4. Discussion

During the first wave of the COVID-19 pandemic, a total of 168 countries closed their borders, and resettlement movements were suspended, hence aggravating the need for international protection of migrants and refugees [28]. The pandemic harmed the mental health of the global population, including those in the European region, with a disproportionately severe impact on refugees and other migrants [32].

This review identified eleven publications from European countries, reviewing data on the effects of the COVID-19 pandemic on the mental health of international migrants in those countries. The results show a diversity of themes related to the effects of COVID-19 on the mental health of migrants and societal responses at micro, meso, and macro levels. This variety relates not only to the different sociodemographic needs across countries and regions but also to the characteristics of the healthcare systems and the variations in the COVID-19 management and adoption of measures [33].

Findings from the reviewed publications provide valuable insights and contribute to a better understanding of the condition of migrants in the context of the COVID-19 pandemic. First, this review describes mental health outcomes across different European countries during the COVID-19 global pandemic. Second, it informs about the social determinants as the social, physical, and economic conditions that most affect mental health outcomes and resilience factors. Third, these identified factors can inform future policy measures, focused on supporting the most at-risk-identified populations, that is the most socioeconomic vulnerable groups of migrants (those undocumented and those with employment and housing insecurity), and provide a strategy to mitigate special risks in these subpopulations.

Despite the variation in the countries’ response to the coronavirus pandemic, several public health measures focused on the continuity of care for mental health service users and facilitating access to mental health assessment and care for new patients were identified. Retaining existing services while promoting new health equity practices and providing individualized mental health services to patients who already have mental disorders or who have developed them during the pandemic needs to be considered. Telehealth services have been recommended to provide continued mental health care for those with existing mental health conditions, for populations who face barriers to accessing care and to combat the impacts of the pandemic [34].

Health workers at the frontlines in Europe and worldwide were the ones at a greater risk of being infected since the beginning of the outbreak of COVID-19, especially those from migrant and minority ethnic backgrounds, at a higher number and risk because of their migrant and socioeconomic backgrounds [24]. Frontline healthcare workers have faced unprecedented demands during the COVID-19 pandemic that have tested their resilience, needing the implementation of coping strategies at the workplace to support resilience at a personal, professional, and organizational level to safeguard mental health in frontline healthcare professions [35].

There is a need for the implementation of a COVID-19-related mental health monitoring system that includes outcomes related to mental health service use [36].

### 4.1. Strengths and Limitations of the Research

This review has limitations that should be addressed when interpreting the results. First, only three databases were consulted for identifying papers with no additional search strategy, such as searching references of the included papers or references of literature reviews identified during the screening process. A strong point, nevertheless, is that Scopus is among the largest databases, with a wide global and regional coverage of scientific journals. In addition, the search strategy was comprehensive and followed the recommendations made by the Joanna Briggs Institute (JBI) [18], such as the Population/Concept/Context method to identify the main concepts and the definition of inclusion criteria. As a second limitation, only the studies conducted in European countries were chosen to be included in this review, with no inclusion of comparisons with other contexts. Plus, the reviewed studies came from only eight countries, and only one global study was included, therefore this review is not representative of the European region. Further research should be conducted to include new publications and regions. Finally, all studies, independently of which design or quality, were included, which may also be considered a limitation. However, this review aimed to summarize the scientific data regarding the multiple effects of COVID-19 on the mental health and well-being of migrant populations from different continents to Europe.

### 4.2. Recommendations for Research and Action

Most of the studies take place in a specific national context, in which the developments over time, the government measures, the COVID-19 communication, and the populations may have considerable differences, forming eco-systems that may be hard to generalize to other contexts [26]. Further research is needed for a better understanding of the impact of cultural, institutional, and structural factors influencing mental health outcomes due to the COVID-19 pandemic. Global studies such as the ApartTogether survey [21] and comparative studies as the study conducted in Poland and the Netherlands among citizens of the above countries as well as among immigrants residing in the Netherlands [27], should be considered in the future with improved sampling procedures to analyze the contextual factors that might impact mental health inequities. Additionally, future research should focus on protective factors and coping strategies that might mitigate the effect of the risk factors known to negatively impact mental health, in particular among the most disadvantaged populations, such as migrants, homeless people, or with difficult living conditions, people with previous experience of severe traumatic events and mental distress [32].

Nevertheless, the results of this review pointed out the need for more effective comprehensive health coverage in countries that see large populations of undocumented migrants with an emphasis on mental health services to reduce further health consequences and strengthen their capacity for resilience. Additionally, of most importance, findings indicate that regularization policies are relevant to facilitate their access to public social services and healthcare to mitigate the consequences of the COVID-19 crisis [29,30].

Mental health services and professionals need to be aware of the need to consider the family situation in psychiatric outpatients, with special attention to people with a migrant background [31]. Community-based approaches could be key to developing multilingual information campaigns and interventions regarding prevention, access to healthcare, vaccination, and social support during the pandemic [30].

Finally, findings serve to highlight the need for proper housing as a strategy to prevent both COVID-19 and mental distress by revealing that people who have more difficulties in accessing health care and preventive measures against the infection experience deterioration of their mental health and increased discrimination by the host population [32].

## 5. Conclusions

This review summarized the existing research on the multiple factors influencing the COVID-19 pandemic’s effects on the mental health of international adult migrants (including refugees, asylum seekers, and irregular migrants), reviewing existing data on European countries to advise recommendations for migrant-sensitive and migrant-inclusive healthcare, while identifying existing knowledge gaps. Findings can serve to inform on the socioeconomic, cultural, geopolitical, and legal environment diversity that forms the context for people’s lives in different settings and which influences mental health outcomes, including mental health workforce shortages and geographical maldistribution of providers, problems that the pandemic made more acute [37]. They can also suggest a solid foundation for providing training and educational support to mental health providers toward an improved focus on migrants’ needs, and effective communication practices.

## Figures and Tables

**Table 1:** Characteristics of the studies.

Author, Date	Journal, Quartile	Domain/Area of Work	Country(ies)	Population and Sample	Methods	Mental Health Outcomes and Instruments
Aragona et al., 2022 [23]	The International journal of social psychiatry Q1	Psychiatry and mental health	Italy	Migrants in treatment in a Mental Health Unit at an outpatient department (n = 81)	Telephone survey to casually selected adults in treatment before the period of lockdown by the clinicians	-Post-traumatic intrusive thoughts and nightmares (PTSD Scale) -Irritable behavior and angry outbursts, and sleep disturbances -Depressed mood and psychic anxiety (Hamilton Depression Rating Scale)
Aragona et al., 2020 [22]	Public health Q2	Public health, Environmental and Occupational Health	Italy	Patients from a mental health outpatient service in Rome (n = 286); main provenance Africa	Retrospective cross-sectional study with data routinely in medical records	Trends in the number of patients and psychiatric categories using the International Classification of Diseases (ICD)-9
Bujek-Kubas and Mojs, 2021 [27]	International journal of occupational medicine and environmental health Q3	Public health, Environmental and Occupational Health	Poland and the Netherlands	Adults (n = 168) Non-immigrants in Poland (n = 50); Dutch citizens (n = 56); Polish immigrants in the Netherlands (n = 62)	Online survey (via Facebook) 1st study before the pandemic in January 2020 and 2nd study in April–May 2020 with the same survey set	-Positive and Negative Experience Scale -Perceived Stress Scale, and -Satisfaction With Life Scale pandemic
Burton-Jeangros et al., 2020 [29]	Frontiers in Public Health Q2	Public health, Environmental and Occupational Health	Switzerland	Undocumented and recently regularized migrants (n = 117 for the online questionnaire and n = 17 for the interviews)	Cross-sectional mixed methods study nested with a cohort study; online questionnaire followed by phone interviews in a subsample	Satisfaction with life, living conditions in Geneva, and social isolation
Gosselin et al., 2021 [30]	Journal of psychosomatic research Q1	Clinical psychology	France	n = 100 immigrants from sub-Saharan Africa in the Greater Paris area	Community-based cohort before the COVID-19 pandemic, with baseline data on the living conditions and mental health, and follow-up telephone interviews between April and June 2020	Depression severity—Patient Health Questionnaire (PHQ9)
Ming and De Jong, 2021 [26]	Sustainability Q1	Geography, Planning and Development	Netherlands	Chinese immigrants living in the Netherlands (n = 268)	Online survey during the second wave of the pandemic (November–December 2020)	Participants compare their well-being with the time before COVID-19 using an adapted version of the Short Depression-Happiness Scale (SDHS);
Moorthy and Sankar, 2020 [24]	Journal of public health Q1	Public health, Environmental and Occupational health	United Kingdom	Black, Asian, and Minority Ethnicity group (BAME) healthcare workers in Leicestershire (n = 200); 78% born outside of the UK	Cross-sectional survey using 20 questions in an electronic format from 2 May to 17 May 2020	Impact of COVID-19 on mental health- Single question
Moran et al., 2021 [31]	Frontiers in Psychiatry Q1	Psychiatry and mental health	Germany	n = 294 psychiatric patients in an outpatient clinic in Berlin, with a high percentage of patients with a migration background	Multimode survey (telephone interview by clinical staff and self-administered by patients) between April and June 2020	-Lockdown-related distress: fear for loved ones; sleep; physical complaints; anxiety; worsening symptoms; drug consumption; fear of contagion; -Family-related distress: more household tension, more work, more domestic violence
Shen and Bartram, 2021 [25]	European Societies Q1	Geography, Planning and Development	United Kingdom	n = 3778 male, of which 3337 are UK-born and 441 are foreign-born	COVID-19 survey via web-based questionnaires; data from Understanding Society—the UK Household Longitudinal Survey and the first two waves (April and May 2020) of the	Mental well-being, (General Health Questionnaire—GHQ)
Solà-Sales et al., 2021 [28]	Healthcare Q2	Leadership and Management	Spain	n = 245 Spanish non-migrants, Spanish migrants, non-Spanish migrants and refugees	Face-to-face or video call interviews between January and May 2021, through a snowball sampling	-Spanish adaptation of the Depression, Anxiety and Stress Scale (DASS-21); -Spanish adaptation of the Brief Resilient Coping Scale (BRCS), -Attitudes toward the COVID-19 outbreak
Spiritus-Beerden et al., 2021 [21]	International Journal of Environmental Research and Public Health Q1	Health, Toxicology and Mutagenesis	European consortium *	n = 20,742 refugees and migrant participants older than 16 years old (survey respondents lived in 170 countries and originated from 159 countries)	Quantitative online global study, as part of the ApartTogether study, from April 2020 until November 2020 in 37 languages	11 mental-health-related items scale: feelings of depression, anxiety, worries, feelings of loneliness, anger, unpleasant reminders of past traumatic experiences, physical reactions to stress, feelings of irritation, hopelessness, sleeping problems, and substance use

* The ApartTogether Consortium includes Belgium, Denmark, Ireland, United States of America, Sweden, United Kingdom of Great Britain, and Northern Ireland, Portugal, Spain, Greece, Italy, and the Netherlands.

## Data Availability

Not applicable.

## Appendix A. Search Strategy

A full search strategy was adopted, including the following terms in MEDLINE [Title/Abstract]:

**#**	**Title-Abs-Key**	
1	“Mental Health” [Mesh]	47,361
2	“Mental Health Services” [Mesh]	100,594
3	“Burnout, Psychological” [Mesh]	14,540
4	“Resilience, Psychological” [Mesh]	7307
5	Well-being	90,984
6	wellbeing	104,723
7	“well being”	90,984
8	“Mental Disorders”	45,273
9	depress *	504,863
10	“Psychiatric Disease”	2597
11	“Psychiatric Disorders”	38,467
12	anxiety	220,555
13	PTSD	28,500
14	“posttraumatic stress symptoms”	2097
15	“Post-traumatic stress disorder”	13,470
16	“Post traumatic stress disorder”	13,470
17	“stress disorder”	34,090
18	“anxiety disorder”	17,806
19	“depression”	374,407
20	psych *	936,057
21	anxi *	239,594
22	#1–#21	1,562,748
23	“COVID-19” [Mesh]	110,949
24	“SARS-CoV-2” [Mesh]	86,555
25	“COVID19”	153,503
26	“COVID-19”	161,458
27	“novel coronavirus”	10,054
28	“2019nCoV”	1251
29	“severe acute respiratory syndrome coronavirus 2”	18,527
30	“2019 Novel Coronavirus”	1571
31	“Coronavirus Disease 2019”	33,204
32	“Coronavirus Disease-19”	1748
33	“SARS Coronavirus 2”	325
34	#23–#33	179,425
35	“Emigrants and Immigrants” [Mesh]	13,984
36	“Undocumented Immigrants” [Mesh]	448
37	“Ethnic Groups” [Mesh]	164,777
38	migrant *	22,129
39	migrat *	354,552
40	immigrant *	27,513
41	immigrat *	14,425
42	emigrat *	7044
43	“asylum seeker”	371
44	refugee *	12,381
45	ethnic *	160,190
46	race	120,120
47	#35–#46	745,515
48	#22 AND #34 AND #47	697
49	(#48) English	691
50	(#49) ≥ 2020	691

* Was used as the symbol for truncation at the point where the spelling of the word could change.
